# Nanoparticle-Modified 3D-Printed Denture Base Resins: Influence of Denture Cleansers on the Color Stability and Surface Roughness In Vitro

**DOI:** 10.3390/nano14100891

**Published:** 2024-05-20

**Authors:** Mohammed M. Gad, Abdulrahman Khattar, Doha M. Alramadan, Zainab H. Al Dawood, Sujood S. Al Shehab, Rabab H. Al Zaher, Layal Osama Alzain, Soban Q. Khan, Mohamed Y. Abdelfattah

**Affiliations:** 1 Department of Substitutive Dental Sciences, College of Dentistry, Imam Abdulrahman Bin Faisal University, P.O. Box 1982, Dammam 31441, Saudi Arabia; 2 College of Dentistry, Imam Abdulrahman Bin Faisal University, P.O. Box 1982, Dammam 31441, Saudi Arabia; abdulrahman.khattar@gmail.com (A.K.); 2170004599@iau.edu.sa (D.M.A.); 2180003167@iau.edu.sa (Z.H.A.D.); 2180002405@iau.edu.sa (S.S.A.S.); 2180001556@iau.edu.sa (R.H.A.Z.); 2170000877@iau.edu.sa (L.O.A.); 3 Department of Dental Education, College of Dentistry, Imam Abdulrahman Bin Faisal University, P.O. Box 1982, Dammam 31411, Saudi Arabia; sqkhan@iau.edu.sa; 4 Department of Prosthodontics, Faculty of Dentistry, Tanta University, P.O. Box 31512, Tanta 31527, Egypt; mohamed.youssif@dent.tanta.edu.eg

**Keywords:** ZrO_2_ nanoparticles, 3D printing, denture base, translucency

## Abstract

This study aimed to evaluate the influence of denture cleansers on the color, stability, and surface roughness of three-dimensional (3D)-printed denture base resins modified with zirconium dioxide nanoparticles (nano-ZrO_2_). A total of 440 specimens were fabricated using one heat-polymerized resin, and two 3D-printed resins (NextDent and ASIGA). According to the nano-ZrO_2_ content, the specimens for each resin were divided into five groups (0%, 0.5%wt, 1%wt, 3%wt, and 5%wt). Each concentration was divided into four subgroups (n = 10) based on the immersion solution (distilled water, sodium hypochlorite, Corega, and Fittydent) and immersion duration (360 and 720 days). The color changes (∆E_00_) and surface roughness (Ra, µm) of each specimen were measured at different time intervals (base line, 360 days, 720 days) using a spectrophotometer and a non-contact profilometer, respectively. The results were statistically analyzed using ANOVA and a post hoc Tukey’s test (α = 0.05). Sodium hypochlorite showed the highest significant color change of all the denture base resins (*p* < 0.001). The average value of ΔE_00_ for sodium hypochlorite was significantly higher than the values for the other solutions (Fittydent, Corega, and water) (*p* < 0.001). Color stability was significantly affected by immersion time for all types of solutions except Corega (*p* < 0.001). All of the tested immersion solutions (distilled water, sodium hypochlorite, Corega, and Fittydent) showed a significant increase in the surface roughness of all the denture base resins (*p* < 0.05). Surface roughness was substantially increased by immersion time for all types of solution except Fittydent (*p* < 0.001). Denture cleansers can result in substantial color change and affect the surface roughness of unmodified and nanoparticle-modified denture base resins. Therefore, the selection of denture cleanser and appropriate types of material is critical for denture longevity.

## 1. Introduction

Polymethylmethacrylate (PMMA) is the most frequently used material for denture base construction [1,2,3]. However, its low surface properties predispose it to *Candida albicans* (*C. albicans*) attachment, the most prevalent fungal pathogen on denture surfaces, leading to denture stomatitis [1,2]. Therefore, maintaining good denture hygiene is important for a healthy underlying oral mucosa [3]. Denture cleansers (DCs) have been proposed for denture cleansing and maintenance protocols [2,4,5]. An ideal denture cleanser should exhibit biocompatibility, be safe to be used on the denture, and effectively eliminate all deposits [2]. 

The primary drawback of denture cleansers lies in their effect on the physical and mechanical properties of the denture base material [1,2,3,6,7]. These denture cleansers increase surface roughness, which increases the accumulation of microbial plaque and hinders plaque removal [1,2,3,6,7]. Immersing dentures in different cleansing solutions increases surface roughness and can negatively affect the color of denture base resins [1,2,3,6,7]. Moreover, the ability of resin material to absorb liquids or degrade over time may result in staining or color changes with prolonged use [1,2,3,6,7]. 

Different denture cleansers have been suggested and investigated with negative outcomes on the esthetics of removable prostheses. Commercial denture cleaning products are divided into several categories, including neutral peroxides with enzymes, hypochlorite, peroxides, acids, mouth rinses, and crude drugs [3,8]. Corega and Fittydent are popular choices for denture cleaning. Fittydent is known for its ability to reduce *C. albicans* adhesion to denture base materials [3,8,9]. On the other hand, Corega denture cleanser has the ability to remove light stains and deposits from the denture base [3,8,10]. Sodium hypochlorite (NaOCl) is also commonly utilized as a disinfecting agent in denture cleansers [3,8,11]. Variations in color, hardness, and surface roughness were reported with NaOCl immersion [6,12]. Immersion time also has an impact on color change (ΔE), as a slight change in color was reported after 90 days, changing to a noticeable color change after 180 days [6,13].

Another option to conventional methods for denture base fabrication is computer-aided design–computer-aided manufacture (CAD-CAM) technology. It can be classified into two categories: subtractive manufacturing, which uses computer-aided milling; and additive manufacturing, which uses three-dimensional (3D) printing technology [14,15,16,17,18,19,20]. Three-dimensional printing demonstrates several advantages over subtractive manufacturing, such as its ability to produce complex geometries and produce multiple specimens at the same time, which makes it more productive [14,15,16,17,18,19,20]. Moreover, 3D-printed removable prostheses demonstrate several advantages compared to conventional prostheses, such as fewer appointments being required and improved adaptation of the final outcome, as well as the ease of duplication of the prostheses [20]. 

Previous studies have compared the mechanical properties of 3D-printed resin material with those of conventional auto-polymerizing and heat-polymerized denture base materials [21,22]. Heat-polymerized resin outperformed 3D-printed resin in terms of flexural strength, elastic modulus, impact strength, and hardness values, but showed inferior surface roughness [21,22]. While another study demonstrated the low strength of 3D-printed resin [23]. Therefore, different methods to improve resins’ strength were suggested, including changing the printing parameters and/or incorporating reinforcing materials [24].

A recent study investigated the mechanical and physical properties of loading zirconium dioxide nanoparticles (nano-ZrO_2_) in 3D-printed resin, which was successful [25]. Previous studies assessed the effects of nano-ZrO_2_ on the mechanical properties of photopolymer resin 3D printing and reported that adding nano-ZrO_2_ particles enhanced the properties of 3D-printed resins [26,27]. The optimal nano-ZrO_2_ content in the photopolymer resin for 3D printing when printing at a 90° orientation was 3wt.% and at 0° was 0 wt.% [26]. 

Although 3D-printed resin modifications with nano-ZrO_2_ were investigated in previous studies with promising outcomes, no previous studies showed the effect of denture cleansers on the properties of ZrO_2_ nanocomposite 3D-printed resins. Therefore, this study aimed to evaluate the influence of denture cleansers on the color stability and surface roughness of 3D-printed denture base resins modified with nano-ZrO_2_. The tested null hypothesis was that the denture cleansers show no significant impact on the color stability and surface roughness of 3D-printed denture base resins modified with nano-ZrO_2_.

## 2. Materials and Methods

Power analysis is used to determine the study’s sample size. For a research power of 80%, a 5% level of significance, and a margin of error of 5% for this study, using the calculations provided by the World Health Organization, the sample calculation resulted in 10 specimens being required per group with a total of 440 specimens [1]. 

**Figure 1:**
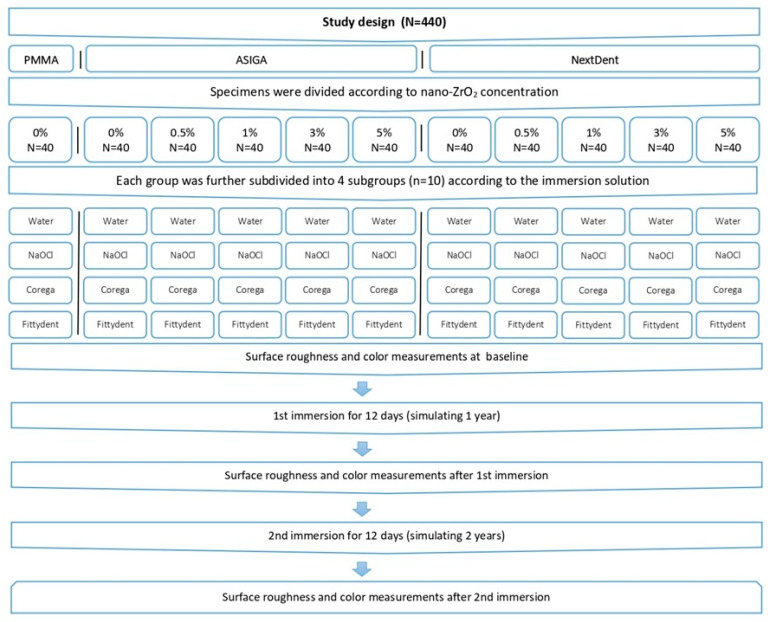
Study design.

One heat-polymerized resin unmodified with nano-ZrO_2_ (negative control group), and two 3D-printed resin materials (“NextDent” and “ASIGA”) (intervention group) were used to fabricate a total of 440 specimens. According to the nano-ZrO_2_ content, specimens were divided into one unmodified group (positive control group) and 4 modified groups (0.5%wt, 1%wt, 3%wt, and 5%wt) (N = 40). Each group was further subdivided into 4 groups (*n* = 10) according to the immersion solution (distilled water, sodium hypochlorite (NaOCl), Corega, and Fittydent) and immersion duration (360 days, 720 days) (Figure 1). 

### 2.1. Preparation of Heat-Polymerized Acrylic Resin Specimens

The exact size of the wax pattern was created by using a square metal mold with dimensions of (10 mm × 10 mm × 2 mm). The wax was placed in the mold in the flasks, after the dewaxing, resulting in a mold for the packing of the acrylic resin. According to the manufacturer’s instructions, the powder and liquid were mixed. After the PMMA specimens were packed, the flasks were prepared to be cured in a thermal polymerization unit [28].

### 2.2. Preparation of Nanoparticles Mixture 

SEM and TEM analyses revealed that the nano-ZrO_2_ had an average granularity of 40 nm and a surface area of 9 m^2^/g [29]. The nano-ZrO_2_ were treated using the silane-coupling agent 3-Trimethoxysilyl propyl Methacrylate as described in a previous study [30]. The silanized nano-ZrO_2_ were weighed using an electronic scale, then added in amounts of 0.5wt%, 1wt%, 3wt%, and 5wt% to 3D-printed resins (NextDent and ASIGA). The resin solutions containing nano-ZrO_2_ were mixed and stirred for 30 min as described in earlier studies [31,32]. The resin was then placed in a 3D mixer machine to be mixed for 120 min. After incorporating the nanoparticles and dividing the resin into multiple bottles with different concentrations, each bottle was shaken for 30 min before proceeding to the printing process [31,32].

### 2.3. Preparation of 3D-Printed Specimens

**Figure 2:**
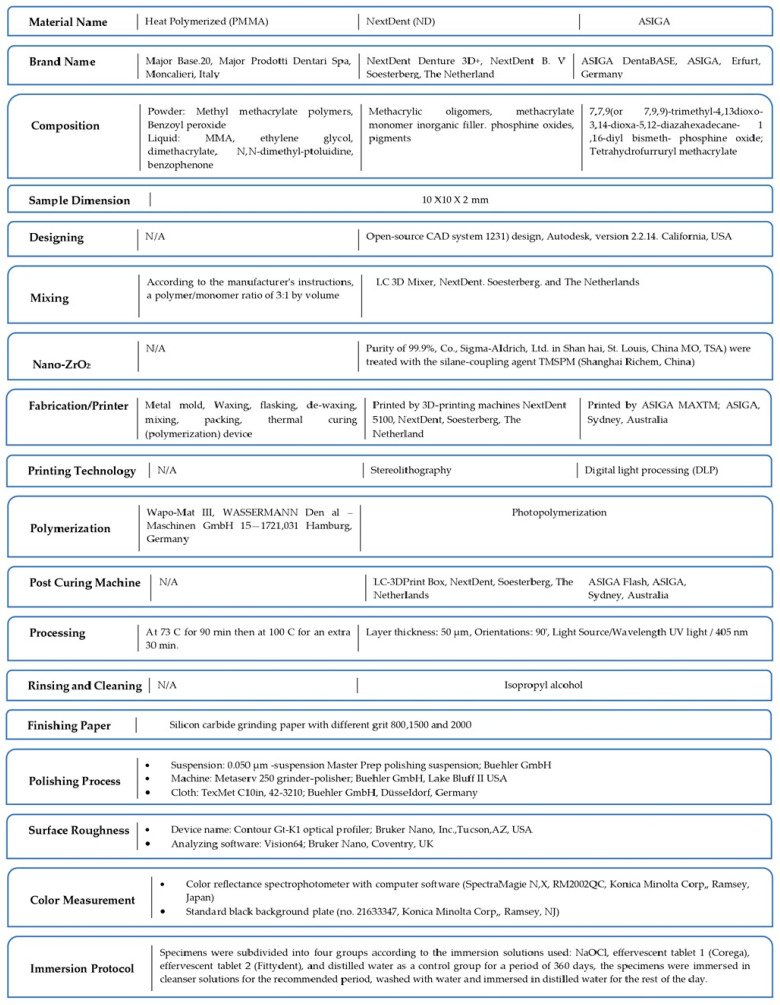
Material specifications and machines used for specimens’ fabrication.

The 3D-printed specimens were designed (10 mm × 10 mm × 2 mm) using an open-source CAD system and saved as a standard tessellation language (STL) file and imported to 3D printers. A printing order was sent to each printer to print the specimens [33]. The printing machine’s specifications, manufacturers, and processing are described in detail in Figure 2. After printing, all the specimens underwent a cleaning process with isopropyl alcohol (99.9%), then were immersed in a bowl of glycerol. According to the manufacturer’s instructions, the post-curing process took 20 min for the ASIGA specimens and 10 min for the NextDent specimens [34]. 

### 2.4. Thermocycling Procedure

A total of 5000 cycles were performed by the thermocycling machine (model MSCT-3, Marcelo Nucci—Me, São Carlos, SP, Brazil) at a temperature between 5 and 55 °C with 30 s of dwell time and 5 s for dripping to simulate the intraoral temperature changes over 6 months [35,36].

### 2.5. Denture Cleanser Preparation and Immersion Protocol 

The different kinds and compositions of the denture cleanser agents utilized in this study and the immersion protocols are summarized in Table 1. Each specimen was suspended and immersed in a solution in a separate container. All jars were labeled to identify the type of solution, stored at room temperature, and the solution was renewed daily. Each subgroup was stocked for 12 days as a standard time to imitate the use of the denture cleanser over 360 days (T1), then stored for another 12 days to simulate use over 720 days (T2) (24 h’s storage time simulated 30 days of using the denture cleanser) [37,38,39,40]. All solutions were prepared by the same operator to minimize discrepancies and deviations in the methodology [37,38,39,40]. 

### 2.6. Testing Procedures

#### 2.6.1. Surface Roughness (Ra)

Surface roughness was measured using a non-contact profilometer. The specimens were dried with absorbent paper and examined for surface roughness (Ra, μm). Ra’s arithmetic average was selected because of its large use, allowing the samples’ surface roughness to be compared with regards to their esthetic aspects [41]. Each specimen was scanned at three different points with a resolution of 0.01 mm. Finally, the averaged surface roughness (μm) for each specimen was calculated using the captured images [22,42]. For all specimens, surface roughness was measured at baseline (T0), 360 days (T1), and 720 days (T2).

#### 2.6.2. Color Measurements

Color measurements were performed on all specimens before exposure to the denture cleanser solutions, considered as baseline readings, using a color reflectance spectrophotometer with computer software. According to the manufacturer’s instructions, before starting any measurement session the colorimeter device was calibrated. All measurements were taken with samples resting on a standard black background plate with background lights turned on. Each specimen was placed in the spectrophotometer’s viewport, and measurements of the L∗, a∗, and b∗ values of each sample were obtained. The measurement process was repeated three times, and the mean values of the L∗, a∗, and b∗ data were calculated [43].

Assessment of the samples after immersion was performed twice. The first assessment was performed after 360 days (T1), followed by a second assessment after 720 days (T2). On the assessment day, the samples were removed from the solutions, then dried; afterwards, the second color evaluation (T1) was performed, as previously mentioned. Then, the third color evaluation was conducted likewise. The color difference values (ΔE_00_) of the materials were calculated (between baseline and different immersion durations) using the CIEDE2000 color difference formula, as described in previous studies [44,45]. In this research, CIEDE2000 values were evaluated concerning both perceptibility and acceptability. Ren et al. [46] documented that the 50% perceptibility threshold was 1.72 CIEDE2000 units, while the 50% acceptability threshold was noted at 4.08 CIEDE2000 units for denture base acrylic resin materials. These thresholds were adopted as benchmarks for perceptibility and acceptability in the present study.

### 2.7. Statistical Analysis 

Mean and standard deviations were computed for the descriptive analysis of the data. The normality of the data was tested by using the Shapiro–Wilk test and insignificant results from the test showed that the data were normally distributed. A parametric test was used for the inferential analysis of the data. A two-independent samples *t*-test was used to compare the means between categorical variables with two categories. One-way ANOVA was used to study the variation in means in relation to the categorical variables with more than two categories. Three-way ANVOA was used to study the interaction effects between time, concentration, and solution. All *p*-values less than 0.05 were considered statistically significant.

## 3. Results

Table 2 demonstrates the ΔE_00_ in relation to time and type of solution used in the study. In PMMA, the variation in the change in color was not statistically significant. However, moving from T1 to T2 increased the ΔE_00_ significantly, and this was observed in each solution except Corega. The mean ΔE_00_ was significantly higher for all types of solution except Corega (*p* = 0.36). For NextDent, significant variation in the color change was found due to the type of solution (*p* < 0.001). In general, the highest color change was observed in NaOCl and the lowest color change was mostly observed in either water or Fittydent. Pairwise comparison showed that for any concentration level at a given point in time, the average value of ΔE_00_ for NaOCl was significantly higher than the values for the other solutions (Fittydent, Corega, and water). In addition, the effect of time was studied at the given concentration of each solution. It was found that the change in color was only significant at the 3% concentration level with water as the solution (*p* = 0.022). Analysis of the data for ASIGA showed that the change in color ΔE_00_ due to the type of solution was found to be significant at the 0% concentration level for both T1 (*p* = 0.000) and T2 (*p* = 0.000). It was also found to be significant at the 1% concentration level when the time was T2 (*p* = 0.021). In these significant results, the maximum color change was observed in either Corega or NaOCl, while the minimum color change was observed in either Fittydent or water. Similarly, the effect of time was also analyzed at given concentration levels and solution types. It was found that the change in color was only significant at the 5% concentration level for the NaOCl solution (*p* < 0.001). 

Analysis of the roughness data showed that in the case of PMMA, at time T1 the variation in roughness due to the type of solution was statistically significant (*p* = 0.000). Similarly, when the variation in roughness was studied in relation to the change in time for each solution, it was found that the variation was significant in each solution except Fittydent (*p* < 0.001). 

Similarly, the effect of the type of solution on roughness was analyzed for each concentration level and time. It was found that the variation in roughness was not significant at the 0% concentration at time T2, the 0.5% concentration level at T0 (control) and T2, and the 5% concentration level at T0 (control) and T2. In addition, analysis of the effect of time on the roughness at each point in time and solution showed that the change in roughness was significant at the 0% concentration level with Corega and water, the 0.5% concentration level with Corega, the 1% concentration level with Corega and water, the 3% concentration level with NaOCl and Fittydent, and the 5% concentration level with water. 

In the case of ASIGA, the variation in roughness in relation to the type of solution was found to be significant at the 0% concentration level at time T2, the 1% concentration level at T0 (control) and T2, the 3% concentration level at T0 (control) and T1, and the 5% concentration level at T0 (control) and T1. Similarly, a study of the effect of time at given concentration levels and solutions showed that at the 0% concentration level and for each solution, the variation in roughness was found to be statistically significant. At the 3% concentration level with NaOCl and Fittydent, the variation in roughness was found to be significant, with *p*-values of 0.003 and 0.004, respectively. At the 5% concentration level with NaOCl as the solution, the variation caused by time on roughness was found to be statistically significant (*p* = 0.002) (Table 3).

The interaction effects of the factors on the color change were studied by using three-way ANOVA (Table 4). It was observed that in NextDent, only the joint effect of solution and concentration was found to be statistically significant (*p* = 0.000). Similarly, in ASIGA, only the combined effect of solution and concentration on the color change was found to be statistically significant (*p* < 0.001).

The interaction effects of the factors on the roughness was studied by using three-way ANOVA (Table 5). It was found that in NextDent, the interacting effects of combinations of two factors (solution and time, solution and concentration, time and concentration) were statistically significant. In addition, the interaction effect of all three factors on the roughness was also found to be statistically significant. Similarly, in the case of ASIGA, the combined effects of pairs of two factors (solution and time, solution and concentration, time and concentration) were statistically significant. However, the combined effect of all factors on the roughness was not statistically significant.

## 4. Discussion

The objective of this study was to evaluate the effect of denture cleansers on the color stability and surface roughness of 3D-printed denture base resins modified with nano-ZrO_2_. The results revealed that denture cleansers significantly affected the color stability and surface roughness of PMMA and 3D-printed denture base resins regardless of the nano-ZrO_2_ concentration. Thus, the null hypothesis was rejected.

To find the matching perceptibility and acceptability threshold of color stability for denture base acrylic resins, the color difference formula that best represents variations between the estimated color and the observed imperceptible to unacceptable color is selected. Color differences were calculated with the International Commission on Illumination (CIE) formulas CIEDE2000 and CIELab, which are most widely used in dentistry [46,47]. The calculated CIELab color difference (DE*) and calculated CIEDE2000 color difference (∆E_00_) have been found to correlate with one another strongly. A previous study showed that when evaluating the PT (the perceptibility threshold) of denture base acrylic resin, the CIEDE2000 equation matches the data better than CIELab and has an equivalent effect on the AT. The higher prediction ability of CIEDE2000 in both PER (perceptibility) and ACC (acceptability) judgments has been demonstrated in previous studies. It is reasonable that CIEDE2000 performs better for PER judgments (even with tiny color differences with PT = 2.52DECMC/1.72DE00) given that it originated from a combined dataset with small color variations (mean: 2.6 DE CIELab units); so, in this study the CIEDE2000 color difference (∆E_00_) formula was selected for color change evaluation to offer a more accurate visual assessment than CIELab for evaluating the denture base acrylic resin’s perceptibility threshold for discoloration [46,47]. 

In this study, the variation in the change in color regarding PMMA was not statistically significant. However, the change in time from T1 to T2 increased the ΔE_00_ significantly, and this was observed in each solution except Corega. Alfouzan et al. [48] reported lower color stability of conventional denture base materials compared to that of a 3D-printed group. According to Ferracane [49], the polarity of PMMA molecules leads acrylic resin to exhibit a propensity for absorbing solvents or water. The color alteration arises from the absorbed liquid diffusing into the polymer network, resulting in hydrolysis and the formation of acrylic zones with distinct optical properties [49]. This could explain why color changes occur even with immersion in distilled water.

To counteract color alterations in dental prostheses, it is imperative to enhance the color stability of materials by incorporating compounds that fortify their resistance to discoloration [50,51]. In a prior investigation, nano-ZrO_2_, silicon dioxide, and titanium dioxide nanoparticles were integrated into PMMA, followed by an assessment of color stability subsequent to immersion in various discoloring beverages [50]. The addition of nano-ZrO_2_ notably amplified the color stability of PMMA, offering a potential avenue for hindering discoloration in denture base resins [50,51]. However, findings from the current study revealed that all denture cleansers induced modifications in both the color and surface roughness of PMMA and 3D-printed nanocomposite denture base resins [50,51].

The denture cleansers, especially those containing peroxides, may cause decomposition and hydrolysis of polymerized acrylic resins, as well as the breakdown of organic pigment compounds, which can lead to color changes in the material [52]. This highlights the importance of choosing appropriate cleansers to maintain the integrity and appearance of dental prosthetics. The choice of denture cleanser should indeed consider various factors including cleanser composition, chemical interactions, concentration, and the duration of immersion [3]. By considering these factors and choosing a denture cleanser that is compatible with the specific denture base resin, at an appropriate concentration, and with the recommended duration of immersion, you can help ensure effective cleaning without compromising the integrity of the denture.

In spite of the advantages of employing NaOCl for disinfection and eliminating biofilm and stains, it comes with drawbacks, including the risk of bleaching [1,2,3]. According to the findings of this study, NaOCl demonstrated the most substantial color transformation, as measured by the ΔE parameter. These results are in agreement with prior investigations, which have consistently indicated NaOCl’s tendency to cause significant alterations in color, especially over prolonged immersion durations [53,54]. Robinson et al. noted that the solvent in denture cleansers permeates the polymer network, causing expansion of intermolecular spaces, resulting in the leaching of internal pigments and infiltration of external colorants, likely leading to color change [54]. When NaOCl comes into contact with certain dyes or pigments within the material, it can lead to decomposition of the chlorine and subsequent degradation of the material. Additionally, the absorption of water-containing chemicals during immersion in NaOCl solutions can further exacerbate material degradation, eventually resulting in noticeable color changes [55]. The effect on color associated with NaOCl is indeed linked with the absorption of the aqueous solution and its active chlorine content by the dental material. NaOCl decomposes, releasing atomic chlorine, which can then react with dyes or pigments present in the material, leading to their degradation and subsequent color change [56]. It is noteworthy that among the test groups, only NaOCl resulted in a significant difference. This underscores the fact that different disinfectants can interact with denture base materials in distinct ways. This variability in interaction highlights the importance of carefully selecting disinfectants based on their compatibility with specific dental materials to minimize adverse effects such as color changes and material degradation [57]. 

Color stability was significantly affected by immersion time for all types of solution except Corega. NaOCl was the denture cleanser that induced significantly more color changes than other cleansers, while surface roughness was significantly affected by immersion time for all types of solution except Fittydent. The surface roughness of the samples was impacted by the immersion time and solution type for both ASIGA and NextDent samples.

In the current study, all combinations of denture cleansers with denture base materials resulted in changes in the color and surface roughness of PMMA and 3D-printed denture base resins regardless of nano-ZrO_2_ concentration. The combined effect of the solution and concentration affected the NextDent and ASIGA samples. The surface roughness values for each group are detailed in Table 3. Increasing surface roughness can lead to infections of the underlying tissues by increasing biofilm adhesion, microbial development, and food residue retention areas that are challenging to clean [58]. Our findings indicated that the denture cleaners have a direct impact on surface roughness. The effect of immersion in different types of solution in comparison to the control group (water), was to increase surface roughness [7,18], and frequent immersion in chemical cleaners markedly increased the surface roughness of an acrylic base material according to Duyck et al. [59] and Pinto et al. [60]. The change in surface roughness caused by plasticizers leaking out as a result of denture cleaners and conventional heat curing is due to reduced structural crosslinking [6].

NaOCl caused an increase in surface roughness for all 3D-printed denture base materials compared to the control group (water), irrespective of the selected type of denture base material and immersion, which aligned with previous studies [1,61], while Ozyilmaz et al. reported that water sorption after immersion in water increased surface roughness. A previous study showed that the surface roughness of acrylic resin specimens immersed in NaOCl had no significant changes [62]. Compared to other denture cleansers, NaOCl showed greater surface roughness alterations in this study. This contrasts with prior investigations [3]. NaOCl has a bactericidal effect that acts directly on the organic matrix of the plaque, dissolving the polymer structure and removing the plaque from the denture while increasing the roughness of the surface [63].

There is significant changes between samples before immersion (T0) and after immersion (T1, T2) regardless of different immersion types. The use of denture cleansers resulted in decreased surface roughness over time. When an acrylic resin is immersed for a longer period of time, more water molecules are absorbed. These molecules remain within the spaces between polymer chains and function as plasticizers, weakening the acrylic resin denture base [64]. However, according to Ana Lucia Machado, roughness increased (from 0.12 to 0.26 μm) during two cycles of chemical cleaning. The materials DuraLiner II and Kooliner have much rougher surfaces after repeatedly being soaked in sodium perborate for disinfection. One possible explanation for the increased roughness of Kooliner and DuraLiner II resins could be the higher level of residual monomer in the surface layer of auto-polymerized acrylic resins. The second explanation for the mechanical cleaning action and increase in surface roughness is the bubbling caused by oxygen release [53].

In this current study, it has been observed that all denture cleansers exhibit an impact on the samples under investigation, irrespective of the material being examined and the concentration of nanoparticles present within it. From a clinical point of view, this study demonstrated that the immersion of dentures in denture cleansers could increase the surface roughness and cause color changes. Increased surface roughness facilitates bacterial adhesion and colonization, resulting in color change. This suggests that the effectiveness of the cleansers is consistent across different materials and nanoparticle concentrations in the study. Nonetheless, depending on the length of immersion, concentration, and chemistry of the resin–cleaner combination, clinicians should choose the appropriate denture cleanser for each type of denture material.

This study has several limitations that should be acknowledged. Firstly, the in vitro design may not fully replicate the complex clinical conditions found in the oral cavity. Factors such as nutritional habits, oral hygiene practices, saliva quality, and pH fluctuations can all influence the outcomes in a real-world setting. Additionally, the specimens used in this study did not accurately mimic the configuration of dentures in the mouth. Moreover, further surface roughness measurement parameters should be used, such as Rz (mean roughness depth), Rq (root mean square), and Rsk (roughness skewness). Future studies should aim to address these limitations by conducting studies using different 3D-printed materials fabricated in a denture configuration and evaluating the surface roughness using several measurement parameters. Such studies could expose the specimens to mechanical and thermal stresses that closely resemble those encountered in the intraoral environment. This approach would provide a more comprehensive understanding of how denture materials perform under realistic conditions and help to guide clinical practice more effectively. Furthermore, more mechanical and tribological properties could be measured in future studies.

## 5. Conclusions

Denture cleansers have an impact on the color changes in PMMA and nanocomposite denture bases regardless of nano-ZrO_2_ concentration and denture base resin type. NaOCl caused the maximum color change on PMMA and all nanocomposite denture base resins when compared to the other solutions (Fittydent, Corega, and water). Color stability is significantly affected by immersion time for all types of solution except Corega, while surface roughness is significantly increased by immersion time for all types of solution except Fittydent. NaOCl is not recommended as a denture cleanser, while other solutions could be recommended when the denture base is additively fabricated using 3D-printed nanocomposite resins. 

## Figures and Tables

**Table 1:** Immersion preparation and immersion protocol.

Denture Cleanser	Description/Manufacturer	Composition	Immersion Solution Preparation and Instructions	Simulation Immersion Protocol
Sodium hypochlorite NaOCl (S)	Sodium hypochlorite solution	5.25% sodium hypochlorite solution	(i) A solution of 5.25% NaOCl was diluted by combining 50 mL of NaOCl with 200 mL of water to achieve a concentration of 1% NaOCl. (ii) Immersion for 10 min at room temperature was performed.	(i) Baseline measurement (T0) was conducted by immersing for two days in distilled water. (ii) Next, all specimens were immersed for 12 days in solutions, mimicking one year of immersion. Subsequently, measurements were conducted again (T1). (iii) Following an additional 12 days of immersion to simulate two years of immersion, measurements were repeated (T2). (iv) Between each immersion, specimens were retrieved, rinsed with water, and then, immersed in distilled water at room temperature before the subsequent immersion cycle.
Distilled water (DW)	Distilled water	-	Immersed in DW at room temperature throughout experimental time.	
Corega (effervescent tablet 1)	Disinfectant effervescent tablet (Dungarvan, Co. Waterfold, Ireland)	Sodium bicarbonate, Sodium carbonate peroxide, potassium caroate (potassium monopersulfate), sodium carbonate, citric acid, TAED, sodium benzoate, PEG-180, sodium lauryl sulfate, VP/VA copolymer, aroma, subtilisin, cellulose gum, CI 42090, CI 73015, CI 19140	(i) One tablet was dissolved in 200 mL of warm tap water (40 °C). (ii) Immersion for 3 min occurred once per day.	
Fittydent (effervescent tablet 2)	Disinfectant effervescent tablet (Fittydent International GmbH Carlbergergasse, Wein, Austria)	Sodium bicarbonate, potassium monopersulphate, sodium perborate monohydrate, surfactant, form booster, colorant, flavoring agent, excipient	(i) One tablet was dissolved in 200 mL of warm tap water (40 °C). (ii) Immersion for 5 min occurred once per day.	

**Table 2:** Variation in color change ∆E_00_ due to type of immersion solution and immersion time.

Material	%	Time	NaOCl Mean (SD)	Fittydent Mean (SD)	Corega Mean (SD)	Water Mean (SD)	*p*-Value
PMMA		T1	1.5 (0.3)	1.5 (0.6)	1.5 (0.6)	1.4 (0.5)	0.116
		T2	2.4 (0.5)	2.4 (0.6)	1.9 (0.5)	2.2 (0.3)	0.136
	*p*-value		0.000 *	0.003 *	0.135	0.001 *	
NextDent	0%	T1	4.8 (2.0)	0.6 (0.3) ^a,b^	0.8 (0.5) ^a,c^	1.6 (1.6) ^b,c^	0.000 *
		T2	4.6 (0.7)	0.9 (0.4) ^a,b^	1.2 (0.4) ^a,c^	1.7 (1.7) ^b,c^	0.000 *
	*p*-value		0.692	0.071	0.093	0.885	
	0.5%	T1	3.2 (1.2)	0.47 (0.4) ^a,b^	1.2 (1.6) ^a,c^	0.65 (0.6) ^b,c^	0.000 *
		T2	3.8 (0.7)	0.87 (0.8) ^a,b^	0.6 (0.2) ^a,c^	0.81 (0.2) ^b,c^	0.000 *
	*p*-value		0.173	0.170	0.263	0.450	
	1%	T1	4.9 (1.1)	0.55 (0.3) ^a,b^	0.84 (0.5) ^a,c^	0.54 (0.4) ^b,c^	0.000 *
		T2	5.2 (1.1)	0.89 (0.6) ^a,b^	0.65 (0.3) ^a,c^	0.89 (0.4) ^b,c^	0.000 *
	*p*-value		0.552	0.125	0.332	0.055	
	3%	T1	5.24 (0.9)	0.54 (0.4) ^a,b^	0.72 (0.7) ^a,c^	0.38 (0.2) ^b,c^	0.000 *
		T2	5.42 (0.7)	0.77 (0.6) ^a,b^	0.82 (0.5) ^a,c^	0.59 (0.2) ^b,c^	0.000 *
	*p*-value		0.631	0.344	0.727	0.022 *	
	5%	T1	5.41 (0.8)	0.77 (1.2) ^a,b^	1.67 (2.4) ^a,c^	0.66 (1.1) ^b,c^	0.000 *
		T2	5.71 (0.7)	0.94 (1.3) ^a,b^	1.69 (2.2) ^a,c^	0.60 (0.5) ^b,c^	0.000 *
	*p*-value		0.397	0.764	0.981	0.880	
ASIGA	0%	T1	0.63 (0.36) ^a,b^	0.54 (0.54) ^a,c^	0.85 (0.85) ^b,c^	1.71 (0.84)	0.000 *
		T2	0.61 (0.25) ^a,b^	0.63 (0.63) ^a,c^	0.83 (0.83) ^b,c^	2.1 (0.70)	0.000 *
	*p*-value		0.86	0.68	0.95	0.33	
	0.5%	T1	0.81 (0.6)	0.54 (0.2)	0.65 (0.2)	0.49 (0.2)	0.185
		T2	1.13 (0.7)	1.0 (1.0)	0.64 (0.4)	0.58 (0.2)	0.185
	*p*-value		0.27	0.17	0.91	0.35	
	1%	T1	0.73 (0.6)	0.79 (0.3)	0.52 (0.3)	0.36 (0.2)	0.083
		T2	1.1 (0.8) ^a,b^	0.58 (0.5) ^a,c,d^	0.63 (0.3) ^b,c,e^	0.43 (0.2) ^d,e^	0.021 *
	*p*-value		0.24	0.29	0.38	0.39	
	3%	T1	0.54 (0.4)	0.80 (0.5)	0.76 (0.8)	0.77 (0.6)	0.775
		T2	0.73 (0.7)	0.87 (0.4)	0.85 (0.2)	1.1 (0.7)	0.482
	*p*-value		0.49	0.75	0.75	0.25	
	5%	T1	0.53 (0.2)	0.57 (0.6)	0.99 (0.9)	0.78 (1.3)	0.604
		T2	1.19 (0.3)	1.25 (1.4)	0.69 (0.3)	0.49 (0.3)	0.095
	*p*-value		0.000 *	0.187	0.36	0.513	

* Statistically significant at 0.05 level of significance. Same lowercase letters in each row show statistical insignificance.

**Table 3:** Variation in surface roughness (Ra, µm) due to type of immersion solution and immersion time.

	%		NaOCl Mean (SD)	Fittydent Mean (SD)	Corega Mean (SD)	Water Mean (SD)	*p*-Value
PMMA		Control	0.46 (0.1) ^A^	0.43 (0.02)	0.46 (0.05) ^A^	0.45 (0.03)	0.621
		T1	0.73 (0.2)	0.48 (0.14) ^a,b^	0.60 (0.06) ^a^	0.034 (0.06) ^b^	0.000 *
		T2	0.37 (0.02) ^A^	0.41 (0.04)	0.41 (0.04) ^A^	0.039 (0.04)	0.170
	*p*-value		0.000 *	0.249	0.000 *	0.000 *	
NextDent	0%	Control	0.57 (0.05) ^a,b^	0.55 (0.08) ^a,c^	0.59 (0.09) ^A,b,c^	0.74 (0.09)	0.000 *
		T1	0.57 (0.13) ^a,b^	0.59 (0.09) ^a,c^	0.76 (0.09)	0.58 (0.1) ^A,b,c^	0.001 *
		T2	0.58 (0.05)	0.61 (0.09)	0.61 (0.1) ^A^	0.60 (0.13) ^A^	0.894
	*p*-value		0.928	0.358	0.001 *	0.005 *	
	0.5%	Control	0.64 (0.19)	0.57 (0.08)	0.65 (0.05)	0.58 (0.07)	0.240
		T1	0.53 (0.07) ^a,b^	0.64 (0.13) ^c^	0.59 (0.05) ^a,c,d^	0.49 (0.08) ^b,d^	0.003 *
		T2	0.52 (0.04)	0.55 (0.06)	0.48 (0.02)	0.53 (0.1)	0.09
	*p*-value		0.054	0.086	0.000 *	0.132	
	1%	Control	0.64 (0.2)	0.42 (0.03) ^a,b^	0.51 (0.05) ^A,a,c^	0.43 (0.04) ^b,c^	0.000 *
		T1	0.73 (0.2) ^a^	0.48 (0.1) ^b,c^	0.6 (0.06) ^a,b^	0.34 (0.06) ^A,c^	0.000 *
		T2	0.69 (0.1)	0.48 (0.1) ^a^	0.52 (0.06) ^A,a^	0.34 (0.06) ^A^	0.000 *
	*p*-value		0.528	0.351	0.002 *	0.001 *	
	3%	Control	0.63 (0.07) ^A,a^	0.69 (0.05) ^a^	0.49 (0.13) ^b^	0.43 (0.09) ^b^	0.000 *
		T1	0.60 (0.1) ^A,a^	0.8 (0.06) ^A^	0.56 (0.09) ^a^	0.39 (0.05)	0.000 *
		T2	0.43 (0.06) ^a,b^	0.78 (0.07) ^A^	0.46 (0.07) ^a^	0.37 (0.03) ^b^	0.000 *
	*p*-value		0.000 *	0.002 *	0.110	0.182	
	5%	Control	0.47 (0.04)	0.46 (0.05)	0.46 (0.04)	0.48 (0.05)	0.698
		T1	0.54 (0.13)^a^	0.38 (0.08) ^b^	0.47 (0.13) ^a,c^	0.38 (0.04) ^A,b,c^	0.002 *
		T2	0.49 (0.08)	0.46 (0.12)	0.47 (0.15)	0.37 (0.56) ^A^	0.074
	*p*-value		0.219	0.078	0.928	0.000 *	
ASIGA	0%	Control	0.53 (0.1)	0.48 (0.11)	0.52 (0.1)	0.58 (0.07)	0.214
		T1	0.34 (0.09) ^A^	0.35 (0.03) ^A^	0.29 (0.03) ^A^	0.27 (0.08) ^A^	0.051
		T2	0.3 (0.04) ^A,a,b^	0.31 (0.03) ^A,a,c^	0.23 (0.04) ^A,d^	0.28 (0.04) ^A,b,c,d^	0.000 *
	*p*-value		0.000 *	0.000 *	0.000 *	0.000 *	
	0.5%	Control	0.45 (0.08)	0.47 (0.09)	0.54 (0.06)	0.53 (0.08)	0.052
		T1	0.54 (0.09)	0.59 (0.14)	0.57 (0.06)	0.55 (0.07)	0.721
		T2	0.52 (0.08)	0.54 (0.09)	0.53 (0.06)	0.53 (0.06)	0.908
	*p*-value		0.082	0.076	0.452	0.647	
	1%	Control	0.39 (0.13) ^a,b^	0.43 (0.11) ^a^	0.61 (0.08) ^c^	0.57 (0.13) ^b,c^	0.000 *
		T1	0.52 (0.18)	0.53 (0.17)	0.69 (0.09)	0.63 (0.12)	0.05
		T2	0.39 (0.12) ^a^	0.51 (0.09) ^a,b^	0.63 (0.06) ^b,c^	0.67 (0.13) ^c^	0.000 *
	*p*-value		0.086	0.209	0.087	0.18	
	3%	Control	0.61 (0.05) ^A,a,b,c^	0.59 (0.03) ^A,a,d^	0.56 (0.06) ^b,e^	0.65 (0.08) ^c,d,e^	0.021 *
		T1	0.72 (0.07) ^a,b^	0.7 (0.07) ^B,a,c^	0.59 (0.04) ^d^	0.68 (0.11) ^b,c,d^	0.005 *
		T2	0.64 (0.08) ^A^	0.63 (0.09) ^A,B^	0.61 (0.03)	0.66 (0.11)	0.567
	*p*-value		0.003 *	0.004 *	0.106	0.736	
	5%	Control	0.45 (0.09) ^A,a,b,c^	0.34 (0.18) ^a,d^	0.47 (0.03) ^b,d,e^	0.50 (0.07) ^c,e^	0.014 *
		T1	0.59 (0.08) ^B,a,b^	0.38 (0.21) ^c,d^	0.52 (0.05) ^a,c,e^	0.53 (0.05) ^b,d,e^	0.000 *
		T2	0.53 (0.06) ^A,B^	0.45 (0.13)	0.52 (0.05)	0.53 (0.07)	0.160
	*p*-value		0.002 *	0.372	0.05	0.528	

* Statistically significant at 0.05 level of significance. Same lowercase letters in each row show statistical insignificance. Same uppercase letters in each column show statistical insignificance.

**Table 4:** Interaction effects of the factors on the color change on each material.

Material	Source	Type III Sum of Squares	Df	Mean Square	F-Value	*p*-Value
NextDent	Intercept	1389.165	1	1389.165	1421.548	0.000 *
	Solution * time	1.587	3	0.529	0.541	0.654
	Solution * concentration	58.414	12	4.868	4.981	0.000 *
	Time * concentration	0.130	4	0.032	0.033	0.998
	Solution * time * concentration	5.096	12	0.425	0.435	0.949
	Error	351.799	360	0.977		
	Total	3013.228	400			
ASIGA	Intercept	253.478	1	253.478	700.277	0.000 *
	Solution * time	1.570	3	0.523	1.445	0.229
	Solution * concentration	28.403	12	2.367	6.539	0.000 *
	Time * concentration	0.255	4	0.064	0.176	0.951
	Solution * time * concentration	5.358	12	0.447	1.234	0.258
	Error	130.309	360	0.362		
	Total	427.931	400			

* Statistically significant at 0.05 level of significance.

**Table 5:** Interaction effects of the factors on the roughness of each material.

Material	Source	Type III Sum of Squares	Df	Mean Square	F-Value	*p*-Value
NextDent	Intercept	174.928	1	174.928	20,012.946	0.000 *
	Solution * time	0.472	6	0.079	9.006	0.000 *
	Solution * concentration	2.990	12	0.249	28.507	0.000 *
	Time * concentration	0.199	8	0.025	2.843	0.004 *
	Solution * time * concentration	0.542	24	0.023	2.583	0.000 *
	Error	4.720	540	0.009		
	Total	186.702	600			
ASIGA	Intercept	159.114	1	159.114	18,074.412	0.000 *
	Solution * time	0.169	6	0.028	3.191	0.004 *
	Solution * concentration	1.185	12	0.099	11.213	0.000 *
	Time * concentration	1.878	8	0.235	26.663	0.000 *
	Solution * time * concentration	0.159	24	0.007	0.751	0.799
	Error	4.754	540	0.009		
	Total	172.064	600			

* Statistically significant at 0.05 level of significance.

## Data Availability

Data are contained within the article.
